# Solitary Fibrous Tumor Revealed by Worsening Clinical Depression: A Case Report from Oman

**DOI:** 10.7759/cureus.9584

**Published:** 2020-08-06

**Authors:** Hiba Al-Harthy, Tamadhir Al-Mahrouqi, Athaari Al-Obaidani, Saif Al-Hashmi, Hassan Mirza

**Affiliations:** 1 Psychiatry and Behavioral Sciences, Oman Medical Specialty Board, Muscat, OMN; 2 General Foundation Program, Oman Medical Specialty Board, Muscat, OMN; 3 Radiology, Oman Medical Specialty Board, Muscat, OMN; 4 Psychiatry, Armed Forces Hospital, Ministry of Defense, Muscat, OMN; 5 Behavioural Medicine, Sultan Qaboos University Hospital, Muscat, OMN

**Keywords:** brain tumor, hemangiopericytoma, psychiatric symptoms, depression, neuropsychiatry, oman, solitary fibrous tumor

## Abstract

Brain tumors are serious pathologies that can lead to significant morbidity and mortality, and early detection and diagnosis may result in favorable outcomes for patients. We present the case of a 27-year-old male who was referred to the Department of Behavioural Medicine of the Sultan Qaboos University Hospital (SQUH), Muscat, Oman, in 2019, due to worsening of his depressive symptoms. Depression was the only initial symptom of the patient’s solitary fibrous tumor, and surgical resection of the tumor followed by radiotherapy resulted in the complete remission of his psychiatric symptoms.

## Introduction

Patients with brain tumors may present with a wide range of signs and symptoms, with frequent clinical presentations such as headache, cognitive dysfunction, and seizures [1]. However, in rare cases, brain tumors may only present with psychiatric symptoms, in which patients may present with mood and anxiety symptoms, psychosis, and behavioral and personality changes [2]. Such a presentation can complicate the clinical presentation and result in a delayed diagnosis or may get misdiagnosed as mental illness [3]. Some primary brain tumors can be life-threatening with a poor prognosis, therefore, early screening and detection is crucial, as diagnostic delays have a detrimental effect on survival and quality of life for patients and their families [4].

## Case presentation

A 27-year-old male was referred to the Department of Behavioral Medicine of the Sultan Qaboos University Hospital (SQUH), Muscat, Oman, in 2019, following the worsening of existing depressive symptoms. The patient had a seven-month history of low mood, social isolation, abulia, and apathy. These symptoms initially presented following a social stressor, as the lady he wanted to marry rejected his proposal and married someone else. During the same period, the patient’s initial symptoms emerged, as he would stay in his room most of the time doing nothing, with minimal social interaction with family, and stopped going to the mosque for prayers. And although he stated that he had a great interest in his job, he developed a lack of motivation or drive to attend his job, with progressive absenteeism, and eventually stopped reporting to work; as a result, his salary was suspended. He lost interest in many of the leisure activities that he used to enjoy such as going out with friends or going to the gym. The patient developed sleeping difficulties, with interrupted sleep throughout the night, early morning awakening, and inability to get back to sleep. He reported low levels of energy, with easy fatiguability, and stopped attending to his basic needs, such as personal hygiene, appropriate clothing, and feeding. He required persuasion to shower and keep himself clean. He had a low mood but did not exhibit any thoughts of self-harm or suicide. There were no associated symptoms of anxiety, mania, or psychosis. He denied substance abuse or alcohol consumption, and there was no history of fever or head trauma. His appetite got poorer as his illness progressed, with significant weight loss. The patient experienced occasional headaches that became more severe with time; it was not associated with aura, nausea, or vomiting. The patient had no family history of mental disorders, and this was his first contact with mental health services. Regarding his personal history, he had achieved normal developmental milestones and completed the general secondary school certificate.

He was assessed as an outpatient and started on an antidepressant as well as cognitive behavioral therapy, but due to poor response to treatment and the severity of his symptoms, he was referred to SQUH for inpatient management. On examination, he presented as a depressed, unkempt man, who looked apathetic with minimal answers and no psychotic symptoms. Neurologically, he had right-sided tremors and exaggerated reflexes with normal tone and power, and no focal neurological deficit. He was fully conscious and oriented, and the rest of the systemic examination was unremarkable.

**Figure 1:**
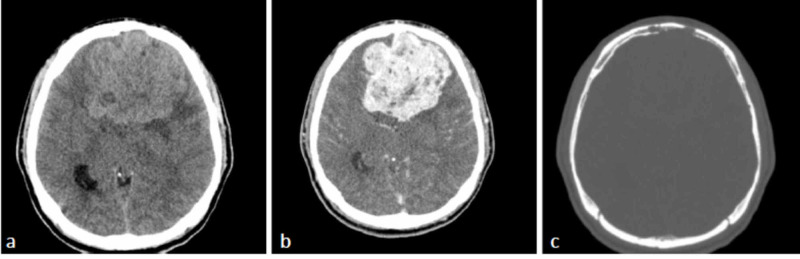
Axial CT scan (a) pre-contrast axial image shows a large heterogeneous mass occupying the basifrontal region spanning across the midline. The mass is hyper-attenuated to the adjacent grey matter. (b) Post-contrast image shows avid enhancement of the mass with non-enhancing necrotic foci. (c) Bone window image shows erosion of the inner endplate of the left frontal bone. CT: computed tomography

**Figure 2:**
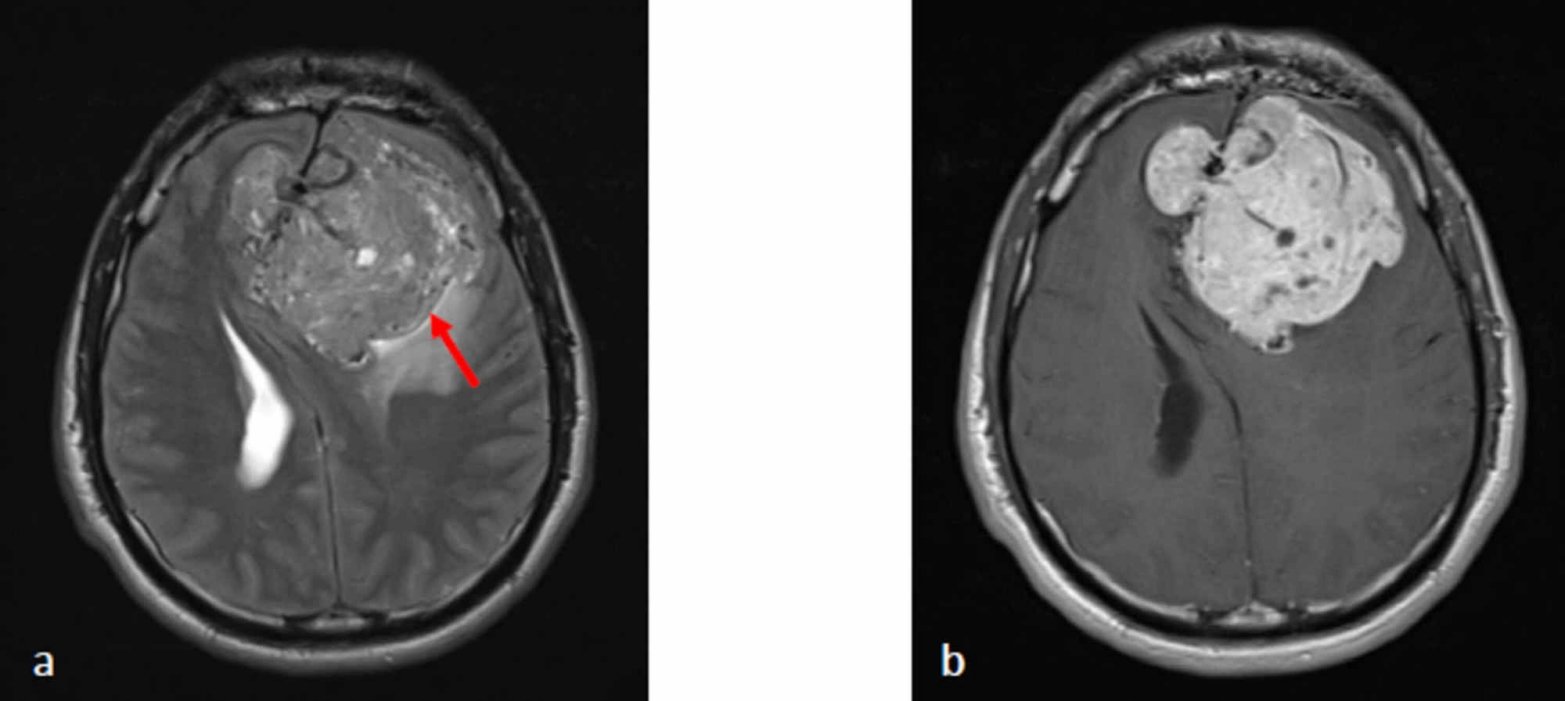
MRI (a) axial T2WI image shows the heterogeneous signal intensity of the mass, which is iso to mildly hypointense to the gray matter with cystic foci and multiple signal voids representing vessels. There is mild perilesional edema with significant mass effect and midline shift to the right. CSF cleft sign (red arrow) is seen denoting the extra-axial origin of the mass. (b) T1 post-contrast image shows an avid enhancement of the mass. MRI: magnetic resonance imaging; CSF: cerebrospinal fluid

The patient underwent pre- and post-contrast CT scan of the brain (Figure 1). The CT scan showed a large extra-axial lobulated midline mass in the basifrontal region spanning across the cerebral falx. The mass measured about 7.0 cm x 7.1 cm x 7.0 cm in the anteroposterior, transverse, and craniocaudal dimensions, respectively. It showed avid heterogeneous signal attenuation and enhancement in post-contrast images with non-enhancing foci denoting necrosis. There was associated perilesional edema and significant mass effect on the frontal horn of both lateral ventricles, which were displaced posteriorly by the mass. There was the erosion of the inner table of the left frontal bone seen on the bone window images. Magnetic resonance imaging (MRI) of the brain before and after the administration of the Gadolinium-based contrast showed the mass with a heterogeneous signal and a predominantly low signal in T1 weighted image (T1 WI) and T2 WI images along with necrotic/cystic areas (Figure 2). Large signal void vessels were seen within the mass, which suggested a highly vascular tumor. No diffusion restriction was appreciated, and nor was a significant blooming artifact to indicate hemorrhage. The mass showed avid heterogeneous enhancement in post-contrast images. The differential diagnoses for such a mass include atypical aggressive meningioma versus hemangiopericytoma (HPC).

**Figure 3:**
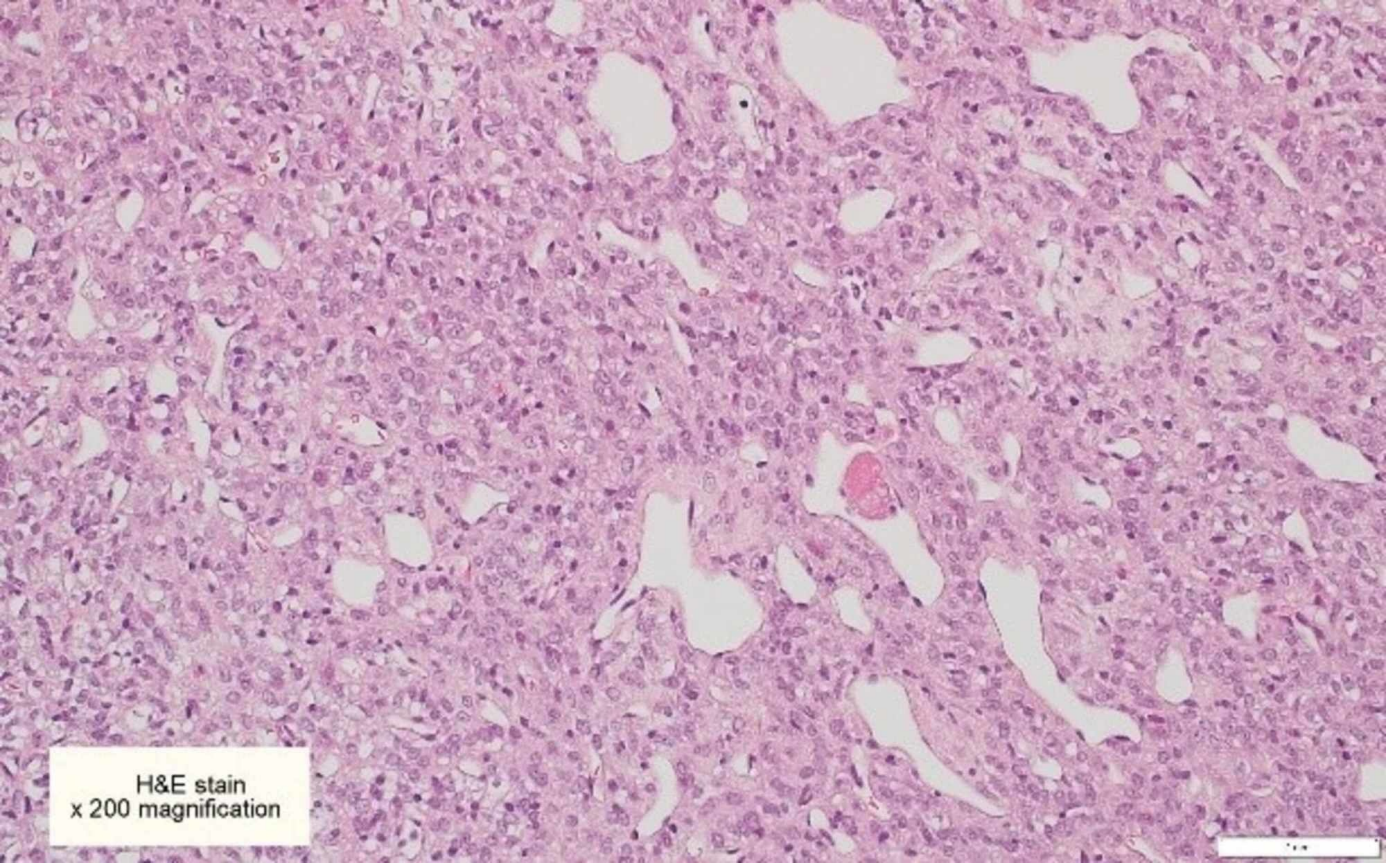
Hematoxylin & Eosin (H&E) stained section of hemangiopericytoma showing closely packed cells that have oval mildly pleomorphic vesicular nuclei, inconspicuous nucleoli, and eosinophilic cytoplasm Note the numerous gaping and occasionally branching blood vessels, some with a “Staghorn” appearance [x200].

**Figure 4:**
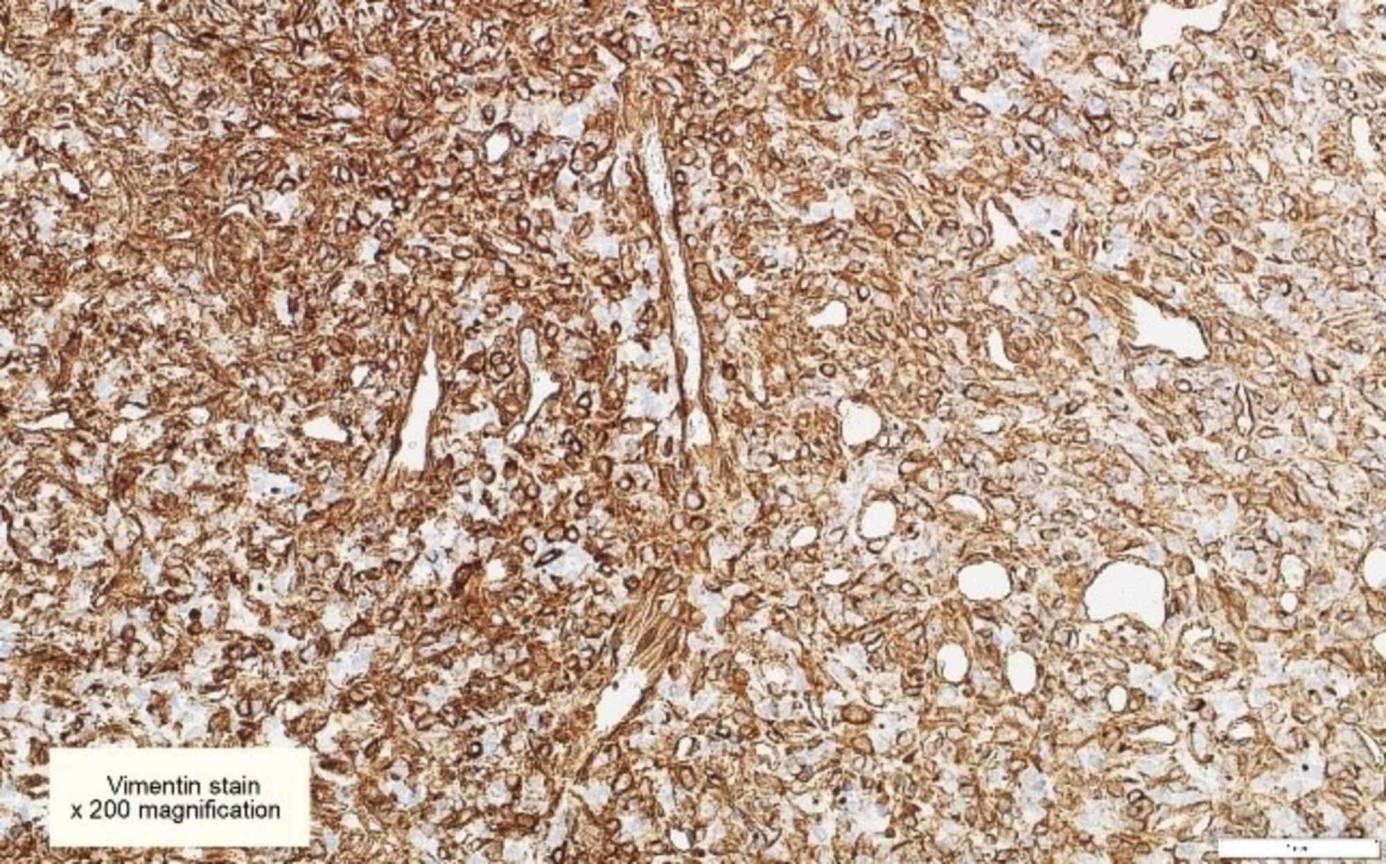
Vimentin immunohistochemical stains are diffusely positive in tumor cells [x200]

**Figure 5:**
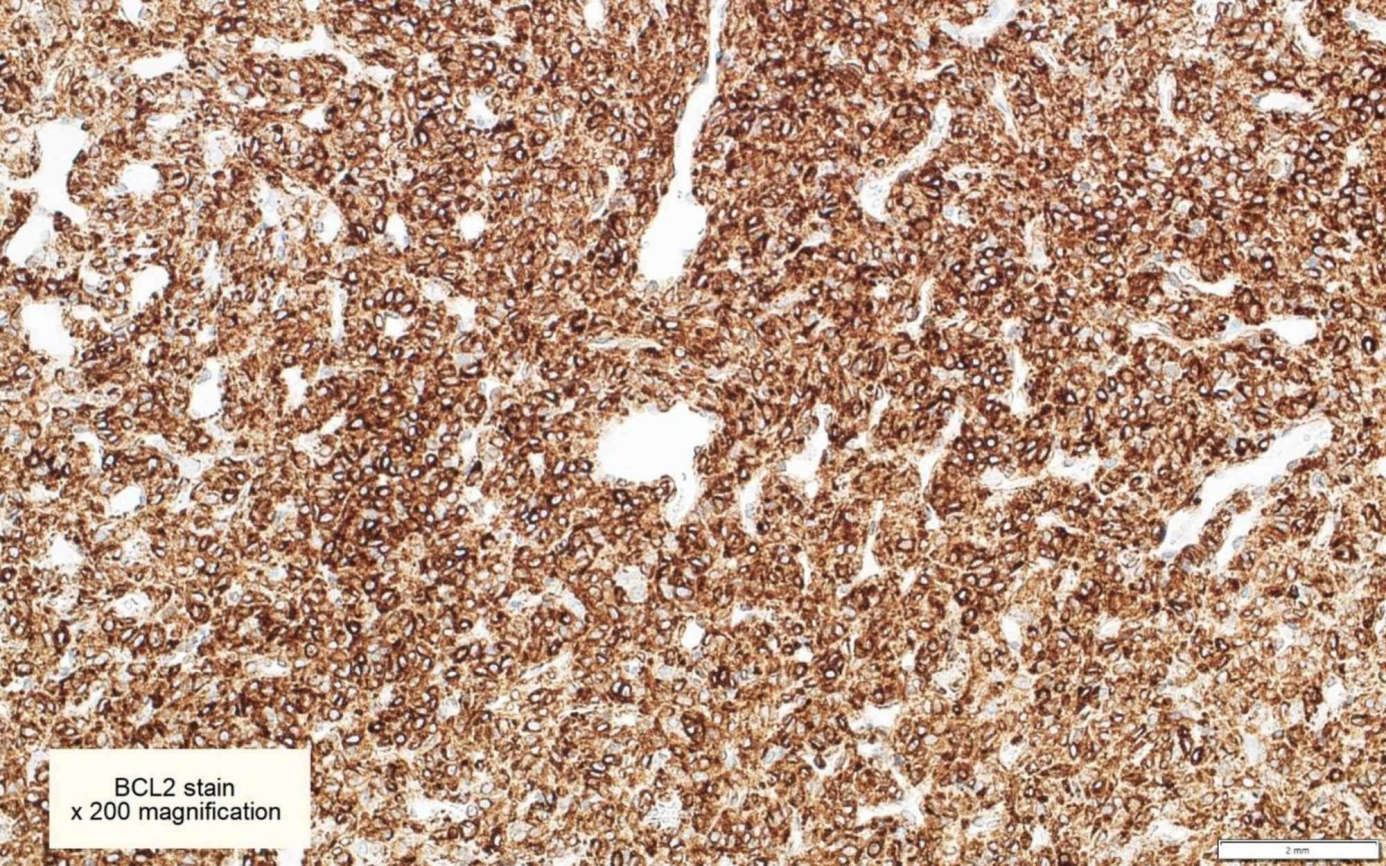
BCL2 immunohistochemical stains are diffusely positive in tumor cells [x200]

**Figure 6:**
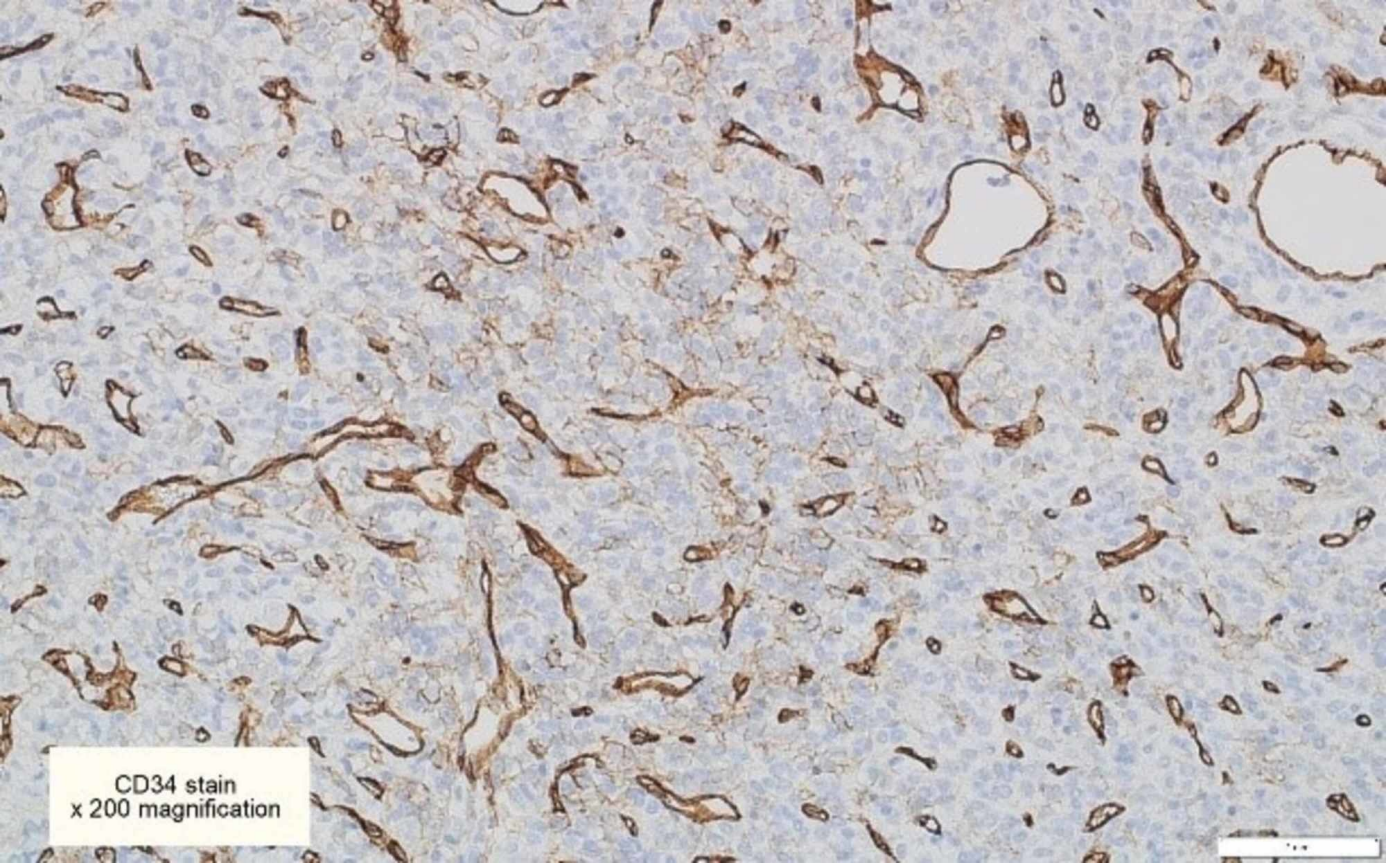
CD34 immunohistochemical stains are focally positive in some tumor cells [x200]

**Figure 7:**
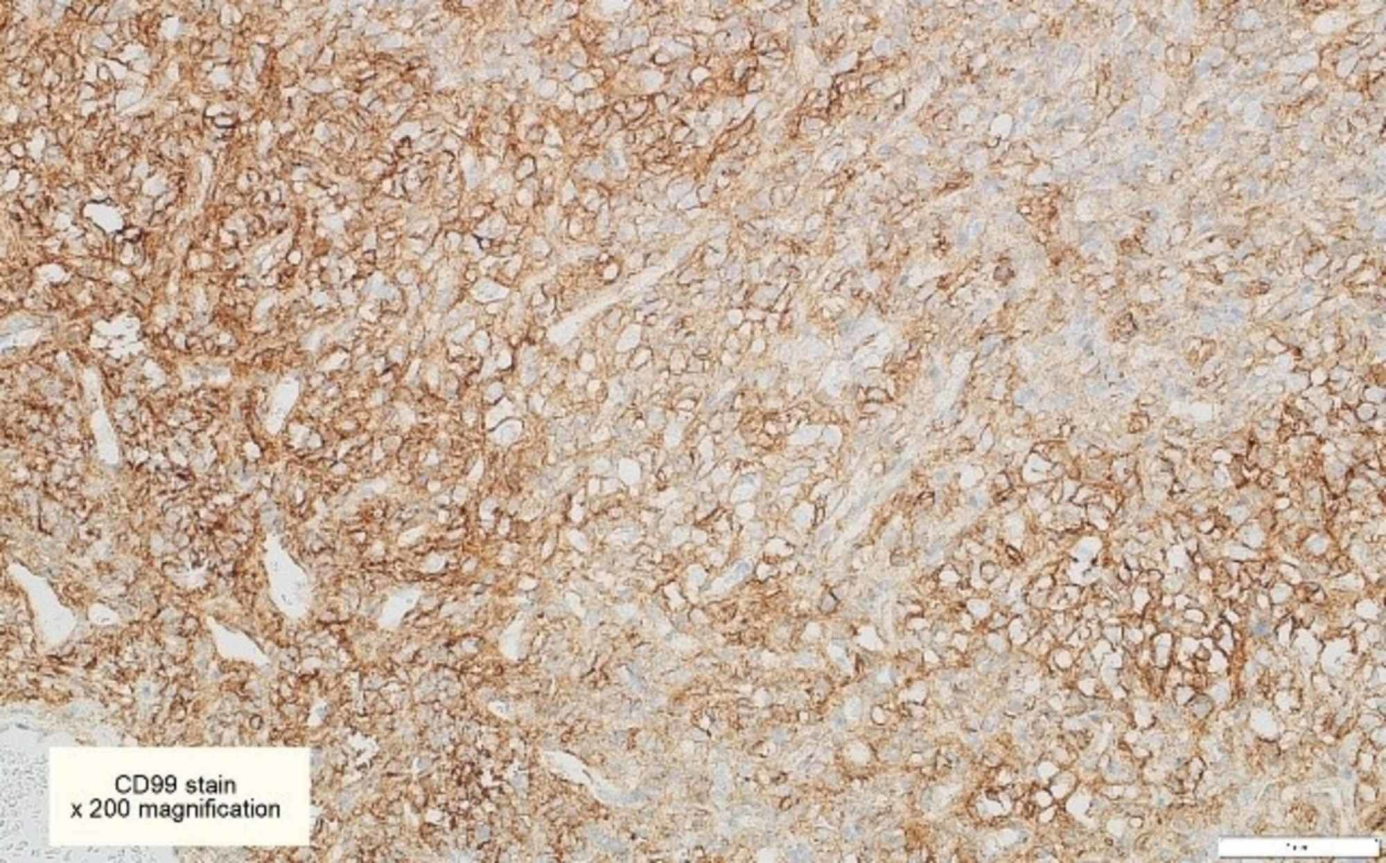
CD99 immunohistochemical stains are focally positive in some tumor cells [x200]

**Figure 8:**
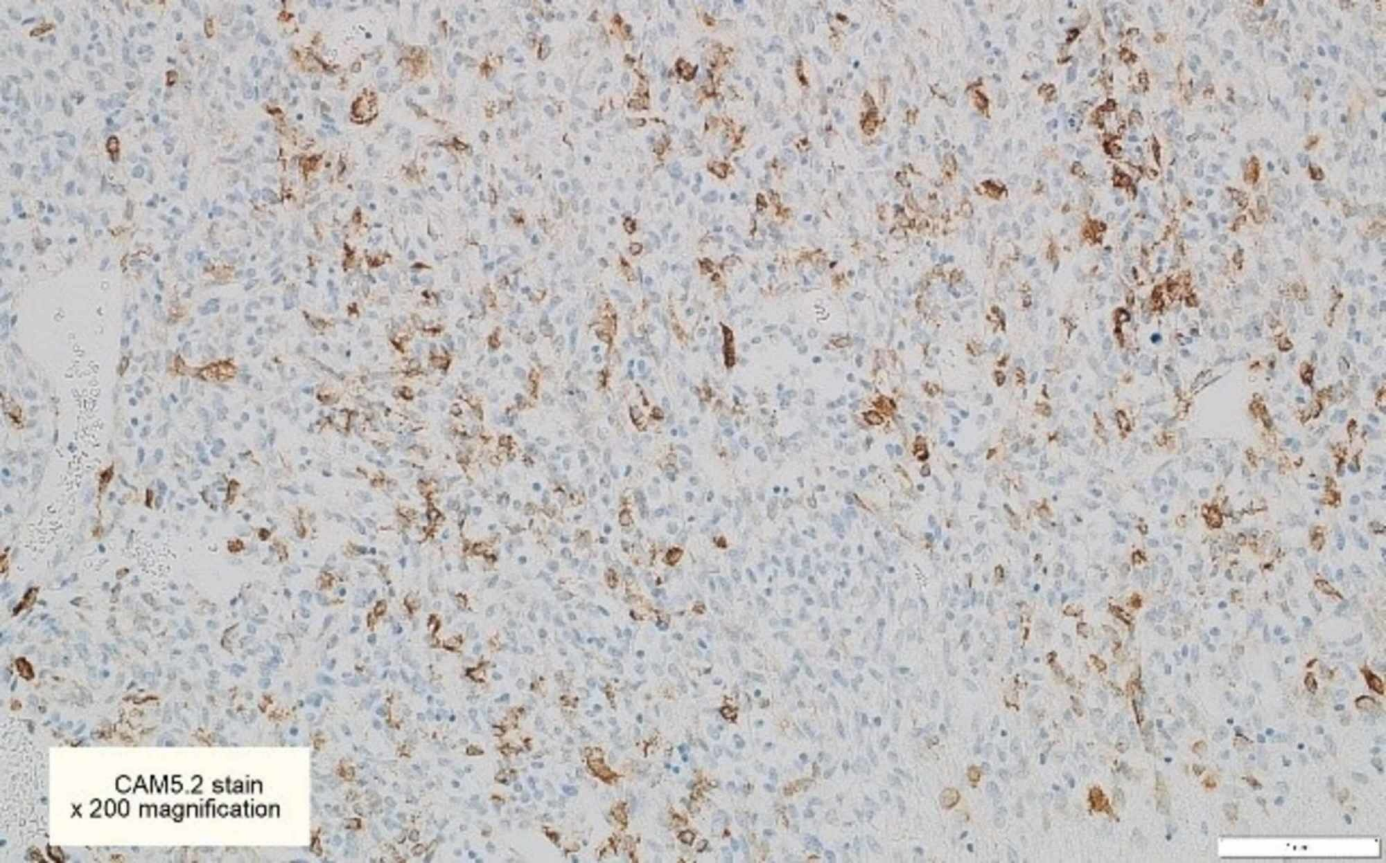
CAM5.2 immunohistochemical stains are focally positive in some tumor cells [x200]

**Figure 9:**
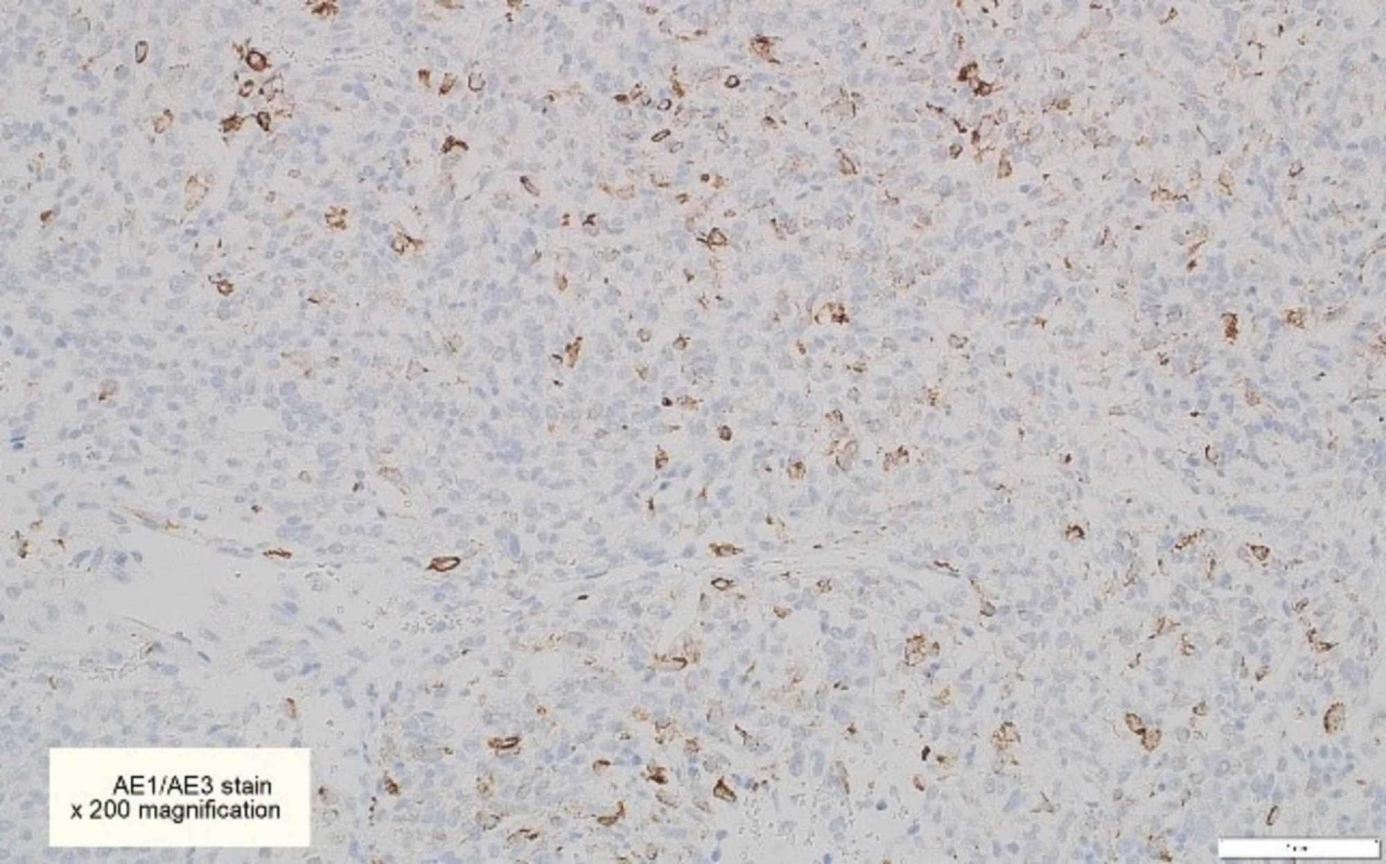
AE1/AE3 immunohistochemical stains are focally positive in some tumor cells [x200]

**Figure 10:**
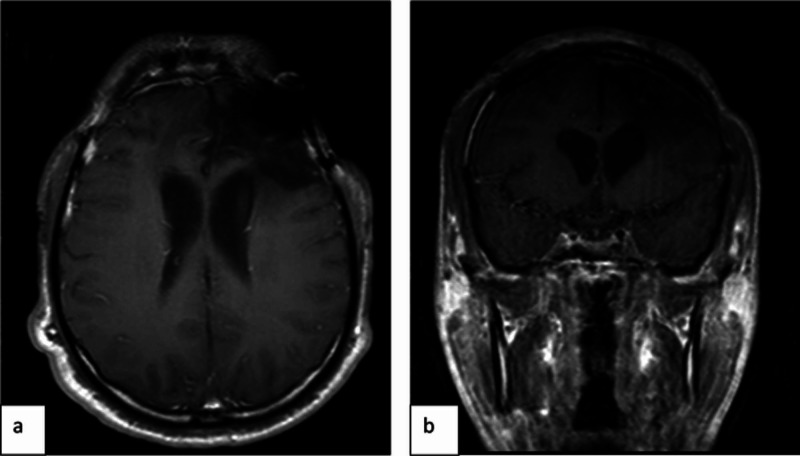
Postoperative MRI brain Post-contrast T1 fat saturation images in axial (a) and coronal (b) planes show edema at the surgical bed with no residual enhancing tumor. MRI: magnetic resonance imaging

The patient underwent stage one bi-frontal craniotomy and biopsy. However, due to extensive blood loss, the procedure was stopped in view of the patient’s safety and to proceed with the gross total resection of the tumor at a second stage. Histologically, the tumor cells showed the ‘staghorn’ appearance, with round to oval-shaped nuclei and inconspicuous nucleoli. There was scattered mitotic activity reaching 3/10 high-power field (HPF), with evidence of bone invasion. Immunohistochemically, the tumor cells were positive for vimentin, B-cell lymphoma 2 (BCL-2), cluster of differentiation 34 (CD34), CD99, CAM5.2, and AE1/AE3. Signal transducer and activator of transcription 6 (STAT6) nuclear expression by immunohistochemistry couldn’t be performed, and NAB2-STAT6 fusion protein could not be checked, as the reagent stock is not available at our center. The first biopsy was suboptimal in view of the marked cautery artifact and definitive World Health Organization (WHO) grading was best deferred to the final excision specimen; the second stage tumor excision was performed abroad, and the histopathology report provided was consistent with hemangiopericytoma, however, further details on WHO grading was not available (Figures 3-9). At the postoperative assessment, the patient was conscious and alert with normal reflexes and neurological motor assessment. Two weeks postoperatively, the patient suffered from severe headaches and complex partial seizures. He then underwent a second bi-frontal craniotomy due to residual tumor, which was successful. Postoperative MRI with contrast showed no enhancement to suggest residual disease (Figure 10). Two months following the surgery, he underwent five radiotherapy sessions, and six months later, a follow-up MRI showed no definite imaging evidence of residual tumor. His condition improved and he recovered well, with normal neurological assessment, he no longer experienced seizures, and there were no depressive symptoms in the follow-up assessments. He resumed work and was back to his pre-morbid level of functioning.

## Discussion

Mood symptoms are prevalent clinical signs in patients with brain tumors [5]. In this case report, we discuss a presentation of worsening clinical depression in a patient diagnosed with hemangiopericytoma (HPC). Intra-cranial HPCs are rare tumors with aggressive behavior, including local recurrence and distant metastasis [6]. In our case, contrast-enhanced positron emission tomography-computed tomography (PET/CT) was performed for staging, and it showed no evidence of distal metastasis. Brain tumors can present with purely psychiatric symptoms with or without neurological manifestations, however, there are no established guidelines for the indications of brain imaging in patients with psychiatric symptoms. A systemic review and meta-analysis by Huang et al. showed that the overall prevalence of depressive symptoms was 21.7% in patients with intracranial tumors [7]. Despite that, there is no consensus on the indication for brain imaging in patients presenting with depressive symptoms; nevertheless, it is recommended in late-onset of depression, treatment-resistant cases, and in patients with a change in neurobehavioral status accompanied by neurological findings [8-9]. HPCs have the tendency to recur and to develop distant metastases many years post-surgical resection. In our case, gross total tumor resection with adjuvant radiotherapy was deemed the treatment of choice [10].

## Conclusions

Brain tumors can present with a wide range of signs and symptoms, and in some cases, psychiatric symptoms are the only presenting feature. Therefore, a thorough history, medical examination, and neurological assessment with a high index of suspicion are important for early diagnosis and intervention. Although there is no consensus or specific guidelines on brain imaging in patients presenting with mood symptoms, neuroimaging should be considered in patients with acute worsening of mood symptoms with associated neurological signs and changes in personality, as well as poor response to conventional psychotropic medications. Such an approach can lead to the early detection of brain tumors in patients presenting with psychiatric symptoms and result in a substantially favorable outcome and prognosis.
